# Proposal of a Standardized Questionnaire to Structure Clinical Peer Reviews of Mortality and Failure of Rescue in Pancreatic Surgery

**DOI:** 10.3390/jcm10061281

**Published:** 2021-03-19

**Authors:** Maximilian Brunner, Franziska Mücke, Melanie Langheinrich, Florian Struller, Felix Rückert, Thilo Welsch, Marius Distler, Stephan Kersting, Georg F. Weber, Robert Grützmann, Christian Krautz

**Affiliations:** 1 Department of General and Visceral Surgery, University Hospital of Friedrich Alexander University Erlangen-Nürnberg (FAU), 91054 Erlangen, Germany; franzi.muecke@freenet.de (F.M.); Melanie.Langheinrich@uk-erlangen.de (M.L.); Florian.Struller@uk-erlangen.de (F.S.); Stephan.Kersting@uk-erlangen.de (S.K.); Georg.Weber@uk-erlangen.de (G.F.W.); Robert.Gruetzmann@uk-erlangen.de (R.G.); Christian.Krautz@uk-erlangen.de (C.K.); 2 Department of Surgery, Medical Faculty Mannheim, University Medical Centre Mannheim, Heidelberg University, 68167 Mannheim, Germany; felix.rueckert@umm.de; 3 Department of Visceral, Thoracic and Vascular Surgery, University Hospital Carl Gustav Carus, 01307 TU Dresden, Germany; thilo.welsch@uniklinikum-dresden.de (T.W.); marius.distler@uniklinikum-dresden.de (M.D.)

**Keywords:** peer review process, causes of death, pancreatic surgery, mortality, complication management, failure to rescue

## Abstract

**Background:** Quality management tools such as clinical peer reviews facilitate root cause analysis and may, ultimately, help to reduce surgery-related morbidity and mortality. This study aimed to evaluate the reliability of a standardized questionnaire for clinical peer reviews in pancreatic surgery. **Methods:** All cases of in-hospital-mortality following pancreatic surgery at two high-volume centers (*n* = 86) were reviewed by two pancreatic surgeons. A standardized mortality review questionnaire was developed and applied to all cases. In a second step, 20 cases were randomly assigned to an online re-review that was completed by seven pancreatic surgeons. The overall consistency of the results between the peer review and online re-review was determined by Cohen’s kappa (κ). The inter-rater reliability of the online re-review was assessed by Fleiss’ kappa (κ). **Results:** The clinical peer review showed that 80% of the patient mortality was related to surgery. Post-operative pancreatic fistula (POPF) (36%) followed by post-pancreatectomy hemorrhage (PPH) (22%) were the most common surgical underlying (index) complications leading to in-hospital mortality. Most of the index complications yielded in abdominal sepsis (62%); 60% of the cases exhibited potential of improvement, especially through timely diagnosis and therapy (42%). There was a moderate to substantial strength of agreement between the peer review and the online re-review in regard to the category of death (surgical vs. non-surgical; κ = 0.886), type of surgical index complication (κ = 0.714) as well as surgical and non-surgical index complications (κ = 0.492 and κ = 0.793). Fleiss’ kappa showed a moderate to substantial inter-rater agreement of the online re-review in terms of category of death (κ = 0.724), category of common surgical index complications (κ = 0.455) and surgical index complication (κ = 0.424). **Conclusion:** The proposed questionnaire to structure clinical peer reviews is a reliable tool for root cause analyses of in-hospital mortality and may help to identify specific options to improve outcomes in pancreatic surgery. However, the reliability of the peer feedback decreases with an increasing specificity of the review questions.

## 1. Introduction

Several studies have found relevant mortality in pancreatic surgery, which entails a need to investigate the underlying causes [1,2,3]. The hospital volume was identified as one of the most important risk factors of perioperative mortality. This effect is mainly based on a significantly lower rate of failure to rescue indicating a more sufficient complication management in high-volume centers [4,5,6,7,8]. Moreover, there is evidence that the experience of the surgeon has a significant influence on perioperative outcomes of pancreatic surgery [9]. As the quality of patient care varies widely among institutions, relevant morbidity and mortality may arise from a variety of other causes that are not detectable by measures of systematic clinical research. In such cases, however, quality management tools provide the possibility to identify the cause of death of individual patient cases. One known tool for the analysis and improvement of medical processes is the peer review process [10,11,12]. The peer review process focuses on the direct exchange of expert knowledge and the mutual assessment at eye level, which helps to generate opportunities for quality and safety improvement in future cases. During the peer review, cases with poor outcome (e.g., in-hospital mortality) are selected and evaluated by other clinicians in order to determine root causes [12]. In the context of complex surgery, such as pancreatic surgery, it is unknown how reliable peer reviews reveal such root causes. In addition, a standardized approach for peer reviews of patient mortality following pancreatic surgery does not yet exist.

The aim of the present study was (1) to identify common categories of underlying (index) complications and causes of mortality in pancreatic surgery, (2) to develop a standardized review questionnaire and (3) to examine, if a peer review with a standardized questionnaire is suitable for root cause analyses of in-hospital mortality.

## 2. Methods

### 2.1. Study Cohort

**Figure 1:**
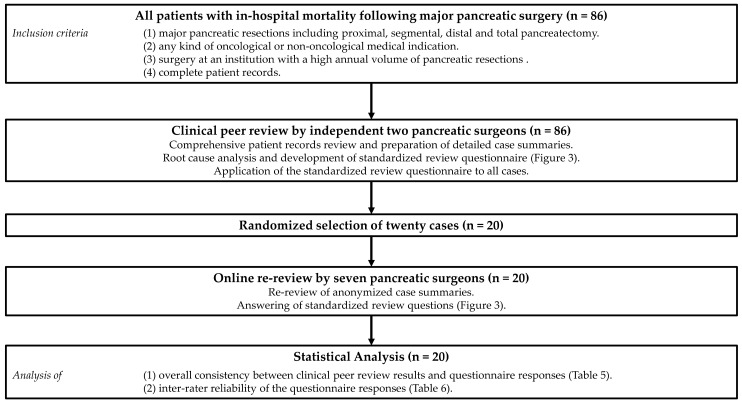
Flow chart showing the course of the study.

All cases of in-hospital mortality following major pancreatic surgery (*n* = 86) at two high-volume centers were included (Figure 1). Major pancreatic surgery was defined as one of the following types of resection: pancreatic head resection, segmental pancreatectomy, distal pancreatectomy and total pancreatectomy with or without additional vascular or adjacent organ resections. The study cohort included patients treated at the Department of Surgery of University Hospital Erlangen between January 2002 and December 2017 (annual volume in 2017: 90 major pancreatic resections; 4 surgeons) and at the Department of Surgery of University Hospital Dresden between January 1994 and December 2014 (annual volume in 2014: 127 major pancreatic resections; 5 surgeons). In accordance with the guidelines for human subject research, approval was obtained from the ethics committee at the Carl Gustav Carus University Hospital (decision number EK 404102018).

### 2.2. Clinical Peer Review

Two pancreatic surgeons that were not involved in patient treatment performed a comprehensive chart review of all included cases (Figure 1). Anonymized case summaries containing necessary clinical data for a mortality review were compiled. For each case with suspected surgical cause of death, a causal chain including at least the underlying (index) complication and the ultimate cause of death was derived. Based on this root cause analysis, definitions for the categories of common index complications and causes of death were established. Subsequently, a questionnaire to standardize mortality reviews in pancreatic surgery was developed. This standardized review questionnaire was applied to all included patient cases. To analyze the delay in recognizing and treating complications, the time between the surgery and the first deviation (any item) from textbook outcome as well as the time between the surgery and the first clinical sign specific to the index complication was determined [13].

### 2.3. Online Re-Review

Using the research randomizer (www.randomizer.org, accessed date: 9 March 2020), 20 cases of the patient cohort were randomly selected to undergo online re-reviews by seven experienced pancreatic surgeons (Figure 1). Theses re-reviews were based on the anonymized case summaries and the standardized review questionnaire. 

### 2.4. Statistics

Data analysis was performed with SPSS software (SPSS, Inc., Chicago, IL, USA). Descriptive data are presented as number (%) or mean [range]. The relationships between surgical index complications and the categories of common surgical index complications and causes of death were graphically illustrated using a Sankey diagram. The distribution of the questionnaire responses according to patient characteristics was assessed by the Chi-square test. Comparisons of metric data were calculated with the Student *t*-test.

To assess the test-retest reliability between the peer review and the online re-review, the majority vote of each questionnaire item of the online re-review had to be determined. Subsequently, the test-retest reliability was examined by Cohen’s kappa (κ). At last, the inter-rater reliability analysis using Fleiss’ kappa (κ) statistics was performed to determine consistency among raters of the online re-review. A *p* value ≤ 0.05 was considered statistically significant.

## 3. Results

### 3.1. Demographics and Characteristics of the Patients

A total of 86 patients (mean age 67 years [range 39–87], 38% female) were included in this study. Demographics and characteristics of the patients are given in Table 1. Most patients suffered from more than one pre-existing diseases (80%) and had an ASA (American Society of Anesthesiologists)-score of three or higher (61%). Indication for surgery was mostly oncological (81%). Surgical procedures included pancreatic head resection (pylorus-preserving pancreaticoduodenectomy (PPPD), Whipple procedure or duodenum-preserving pancreas head resection) (79%), pancreatic left resection (16%) and total pancreatectomy (5%). 26% of the patients received additional vascular resections (68% venous resection, 18% arterial resection and 14% combined resection). 28 patients (33%) had a multivisceral resection including one of the following organs (in 29% more than one organ): bowel (54%), stomach (36%), liver (25%), adrenal gland (18%), kidney (14%).

### 3.2. Clinical Peer Review

**Figure 2:**
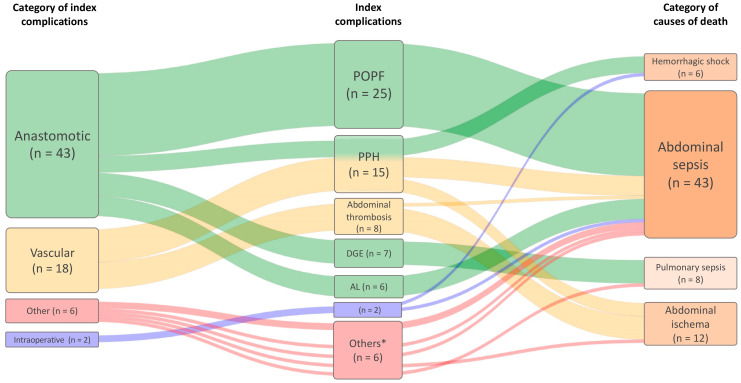
Sankey diagram illustrating the contribution of surgical index complications to causes of death; POPF = post-operative pancreatic fistula, PPH = post-pancreatectomy hemorrhage, DGE = delayed gastric emptying with aspiration, AL = anastomotic leakage (except pancreatic anastomosis), * includes = post-operative pancreatitis (*n* = 2), non-occlusive mesenteric ischemia (*n* = 1), disseminated intravascular coagulopathy (*n* = 1), ileus (*n* = 1), aspiration (*n* = 1).

Mean postoperative time to death was 34 days [range 1–146]. Cause of death was most often related to surgery (80%). In 20% of all cases, patients died from a non-surgical index complication (e.g., myocardial infarction and pulmonary embolism). The relationships and most important contributions between surgical index complications and causes of deaths are visualized in Figure 2. The most common surgical index complications leading to in-hospital mortality were post-operative pancreatic fistula (POPF, 36%), post-pancreatectomy hemorrhage (22%), abdominal thrombosis (12%), delayed gastric emptying causing aspiration (10%) and anastomotic leakage other than pancreatic anastomosis (9%). These surgical index complications were attributed to four categories (62% anastomotic complications, 26% vascular complications, 9% other complications and 2% intraoperative complications). The causes of deaths that resulted from surgical index complications were categorized in abdominal sepsis with organ failure (62%), abdominal ischemia (17%), pulmonary sepsis with organ failure (12%) and hemorrhagic shock (9%), respectively. Non-surgical index complications are listed in Table 2. The first clinical sign specific to the index complication occurred significantly later compared to the first deviation from textbook outcome (*p* = 0.002) (Table 3). This difference was also significant in cases of a surgical cause of death and POPF (*p* = 0.004 respectively *p* = 0.036). In 42% of the cases a delay in recognizing or treating the complication was observed (Table 3). 

**Figure 3:**
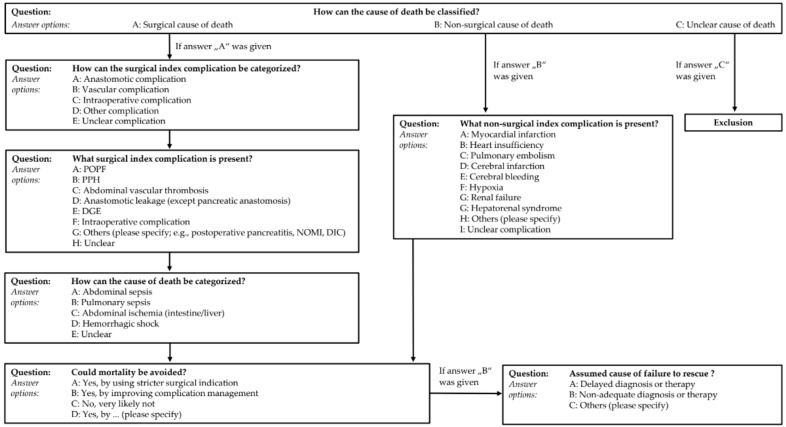
Standardized questionnaire for clinical peer reviews of patient cases with perioperative mortality in pancreatic surgery. POPF = post-operative pancreatic fistula, PPH = post-pancreatectomy hemorrhage, DGE = delayed gastric emptying, NOMI = non-occlusive mesenteric ischemia, DIC = disseminated intravascular coagulation.

The standardized review questionnaire derived from these data is given in Figure 3.

### 3.3. Distribution of Questionnaire Responses According to Patient Characteristics and Surgical Parameters

The distribution of questionnaire responses according to patient characteristics and surgical parameters is given in Table 4; Table 5. Stratification of cases according to preoperative (oncological vs. non oncological, pre-existing diseases <2 vs. ≥2) and surgical parameters (pancreatic head resection vs. pancreatic left resection, vascular vs. no vascular resection, multivisceral vs. no multivisceral resection) showed that patients with more than one pre-existing diseases died earlier (40 vs. 30 days, *p* = 0.050) and more often due to pulmonary septic organ failure and less often due to hemorrhagic shock than those with no or one pre-existing disease (*p* = 0.010) (Table 4). Moreover, the number of patients with a surgical cause of death was significant higher following pancreatic head resections as compared to those with left pancreatectomy (85% vs. 57%, *p* = 0.026). Patients with vascular resection had higher rates of vascular complications (50% vs. 16%, *p* = 0.012) and abdominal ischemia (40% vs. 8%, *p* = 0.012) compared to patients with no vascular resection (Table 5).

### 3.4. Online Re-Review

Out of 20 re-reviewed cases, seven were classified non-surgical and 13 were classified surgical by majority voting. The majority votes for the classification of the cause of death were attained with an average agreement of 76% (non-surgical) and 87% (surgical), respectively. In case of non-surgical classified deaths, majority decisions on the category of common index complications were reached with an average agreement of 73%. The average agreement on the type of index complication and the category of cause of death in surgical classified deaths was 84% and 61%, respectively. Cohen’s κ was run to determine if there was agreement between the peer review and online re-review on all questionnaire items (Table 6). There was a near perfect agreement between the peer review and the online re-review on the category of death (surgical vs. non-surgical; κ = 0.886 (*p* < 0.001); 95% confidence interval (CI 0.670; 1.101)), a substantial strength of agreement on the potential of improvement (κ = 0.765 (*p* = 0.001); 95% CI (0.465; 1.065), the category of common surgical index complications (κ = 0.714 (*p* < 0.001); 95% CI (0.377; 1.051)), the category of cause of death (κ = 0.694 (*p* < 0.001); 95% CI (0.327; 1.061)) as well as non-surgical index complications (κ = 0.793 (*p* < 0.001); 95% CI (0.458; 1.128) and a moderate strength of agreement on surgical index complications (κ = 0.492 (*p* < 0.001); 95% CI (0.178; 0.806)) (Table 6). The inter-rater reliability between all participants of the online re-review assessed by Fleiss’ kappa is given in Table 7. Fleiss’ kappa showed a substantial inter-rater agreement on the category of death (κ = 0.724 (*p* < 0.001); 95% CI (0.581; 0.866)) and a moderate inter-rater agreement on the category of common surgical index complications (κ = 0.455 (*p* < 0001) 95%CI (0326; 0.584)), the category of cause of death (κ = 0.533 (*p* < 0.001); 95% CI (0.311; 0.745)) and surgical index complications (κ = 0.424 (*p* < 0.001); 95% CI (0.329; 0.518)), respectively.

## 4. Discussion

This comprehensive clinical peer review of 86 patients with in-hospital mortality following pancreatic surgery identified four categories of common surgical index complications and causes of death. These categories as well as identified common surgical and non-surgical index complications were incorporated in a standardized questionnaire. Reliability analysis of the questionnaire items found a moderate to substantial test-retest reliability as well as inter-rater reliability.

Pancreatic surgery is associated with relevant morbidity and mortality, which entails the need to investigate the underlying causes [1,2,3]. Several studies have focused on the investigation of mortality rates and the development of prediction models in order to determine the risk of morbidity and mortality in pancreatic surgery. Patient demographics, hospital volume and the expertise of the surgeons have been identified as strong risk factors that affect the perioperative morbidity and mortality rates [1,2,3,4,5,6,7,8,9]. While patient demographics are hard to control, hospital and surgeon volume have been addressed through centralization. Several studies have already reported the benefits of centralization in pancreatic surgery [14,15,16]. As the centralization effect is probably exhausted in numerous countries, additional approaches are necessary to gain further improvements of outcomes. Clinical peer reviews may be an option, but its effect on outcomes is unclear. In addition, such targeted root cause analyses demand a comprehensive analysis of each individual patient case. This study found that surgical index complications accounted for 80% of all deaths. Most of these index complications yielded in sepsis with abdominal organ failure (62%). Data from other studies suggest that at least half of all surgical complications are avoidable [17,18]. In line with an Australian mortality review report, the present data show that a delay in recognizing and treating complications may occur in more than a quarter of deaths [19]. Subsequently, prevention of delayed recognition of complications may improve outcomes.

Pancreatic resections are complex procedures that may result in even more complex post-operative courses as soon as complications arise. In addition, complications originate from and are boosted by preoperative, intraoperative or post-operative errors in patient care. The resulting complexity hampers retrospective root cause analysis including the determination of the final cause of death and the surgical index complications. Therefore, clinical peer reviews will most likely have a low reliability that, in turn, leads to inconsistent proposals of measures to change subsequent practice. This study shows that structuring of the peer review process by means of a standardized questionnaire provides sufficient reliability in regard to index complications, causes of death and potential preventive measures. Of note, the reliability of the peer feedback decreases with an increasing specificity of the review questions (e.g., category of common surgical index complication: ‘anastomotic complication’ vs. surgical index complication: ‘POPF’). This effect probably increases in a multicausal setting that comprises more than one complication and error cause, and subsequently a larger scope of preventive measures. In addition, the inter-rater reliability analysis of the peer re-review showed a lower degree of agreement especially for the last item of the questionnaire (potential of improvement). In view of these facts, it should be noted that the proposed questionnaire has limitations and is not intended to be exhaustive. Especially, specific measures derived from root cause analysis are usually not generalizable due to multifactorial influences in complex clinical settings of individual patient cases. Nevertheless, it should be considered as a tool to structure clinical peer reviews that may prompt in-depth perceptions.

In this study, surgical in-hospital mortality was mainly caused by anastomotic complications. This is in line with the current literature on post-pancreatectomy complications, such as POPF, PPH and DGE. In this regard, the current analysis underlines the relevance of PPH that causes a relevant part of the overall mortality (22%), although it is significantly less common than POPF or DGE. Vascular complications led to in-hospital mortality in a significant part of cases, which corresponds to results of an Australian mortality review report [19]. This category of complications is important to consider and may be due to pre-existing stenoses or inadvertent vascular injury [19]. The latter may be avoided by preoperative anticipation of aberrant vascular anatomy using a novel 3-D visualization technology for post-processing of computed tomography (CT) images, called cinematic rendering [20,21].

This study has limitations that need to be considered. First, the limited sample size and the retrospective design of our study may have incurred some biases. Second, peer reviewing is a subjective assessment and thus prone to bias. Consequently, the standardized questionnaire derived from the clinical peer review in this study should be considered imperfect and improvable. These limitations may be overcome by future investigations with a larger patient cohort in an international, multicenter setting and a prospective study design. Such studies should also include a large panel of international reviewers who are experts in the field pancreatic surgery.

## 5. Conclusions

Patients with in-hospital mortality following pancreatic surgery frequently have long and difficult post-operative courses. The resulting complexity hampers the determination of the final causes of death and the surgical index complications. The use of standardized questionnaires helps to structure the peer review process. The fact that structured clinical peer reviews provide consistent results between different reviewers underlines the usefulness of this quality management tool for root cause analyses of in-hospital mortality following pancreatic surgery. Of note, the overall consistency of the peer feedback decreases with an increasing specificity of the review questions. Further studies are needed to improve the suggested standardized questionnaire and to investigate whether quality enhancement measures derived from clinical peer reviews lead to a sustained improvement of post-operative outcomes.

## Figures and Tables

**Table 1:** Demographics and characteristics of patients with in-hospital mortality after pancreatic surgery.

Demographics		*n* (%) or Mean [Range]
Total number		86
Age (years)		67 [39–87]
Gender	Male	53 (62)
	Female	33 (38)
Body Mass Index (BMI) (kg/m^2^)		25.8 [16.7–37.0]
ASA	II	31 (36)
	III	44 (51)
	IV	5 (6)
	unknown	6 (7)
Alcohol abusus	No	44 (51)
	Yes	27 (31)
	unknown	15 (17)
Smoking	No	55 (64)
	Yes	22 (26)
	Unknown	9 (11)
Pre-existing diseases	<2	38 (44)
	≥2	48 (56)
Metastatic disease	No	79 (92)
	Yes	7 (8)
Neoadjuvant therapy	No	74 (86)
	Yes	12 (14)
Indication of surgery	Oncological	70 (81)
	Non-oncological	16 (19)
Type of surgery	Whipple/PPPD	68 (79)
	Left pancreatectomy	14 (16)
	Total Pancreatectomy	4 (5)
Extented surgery	No	44 (51)
	Vascular resection	14 (16)
	Multivisceral resection	20 (23)
	Vascular and multivisceral resection	8 (9)

**Table 2:** Non-surgical causes of death in patients with in-hospital mortality after pancreatic surgery.

Non-Surgical Index Complication	*n* (%) or Mean [Range]
Myocardial infarction	5 (29)
Pulmonary embolism	3 (18)
Cerebral infarction	2 (12)
Cerebral bleeding	2 (12)
Heart insufficiency	2 (12)
Hepatorenal syndrom	2 (12)
Hypoxia	1 (6)

**Table 3:** Time to first deviation from textbook outcome and reviewer ratings regarding potential of improvement.

	First Deviation from Textbook Outcome * (POD)	First Clinical Sign Specific to Index Complication (POD)	*p*-Value	Potential of Improvement				*p*-Value
				Quality of Indication	Complication Management		None	
					Delay in Diagnosis or Therapy	Non-Adequate Diagnosis or Therapy		
**All patients (n = 86)**	6 [0–34]	12 [0–122]	0.002	9 (11)	36 (42)	7 (8)	34 (40)	-
**Classification of cause of death**								
Surgical (*n* = 69)	5 [0–21]	10 [0–83]	0.004	5 (7)	35 (51)	7 (10)	22 (32)	0.001
Non-surgical (*n* = 17)	8 [0–34]	17 [0–122]	0.165	4 (24)	1 (6)	0 (0)	12 (71)	
**Surgical index complication**								
POPF (*n* = 25)	5 [1–14]	8 [1–29]	0.036	1 (4)	16 (64)	2 (8)	6 (24)	0.362
PPH (*n* = 15)	6 [1–20]	16 [1–83]	0.117	1 (7)	8 (53)	3 (20)	3 (20)	
Abdominal thrombosis (*n* = 8)	4 [1–10]	4 [1–10]	0.351	1 (13)	3 (38)	1 (13)	3 (38)	
DGE (*n* = 7)	8 [0–21]	20 [3–63]	0.186	1 (14)	2 (29)	0 (0)	4 (57)	
Anastomotic leakage ** (*n* = 6)	5 [1–9]	7 [4–9]	0.189	0 (0)	4 (67)	1 (17)	1 (17)	
Intraoperative complication (*n* =2)	0	0	-	1 (50)	0 (0)	0 (0)	1 (50)	
Others (*n* = 6)	7 [1–20]	9 [1–25]	0.325	0 (0)	2 (33)	0 (0)	4 (67)	

* Textbook outcome as defined by van Roessel et al. [13], ** except pancreatic anastomosis, POD = post-operative day.

**Table 4:** Distribution of questionnaire responses according to patient characteristics.

	Oncological *n* = 70	Non-Oncological *n* = 16	*p*-Value	Pre-Existing Diseases <2 *n* = 38	Pre-Existing Diseases ≥2 *n* = 48	*p*-Value
**Time to death (days)**	35 [1–146]	31 [3–62]	0.854	40 [3–146]	30 [1–126]	0.050
**Classification of cause of death**						
Surgical	56 (80)	13 (81)	1.000	32 (84)	37 (77)	0.432
Non-surgical	14 (20)	3 (19)		6 (16)	11 (23)	
**Category of surgical index complication**						
Anastomotic	37 (66)	6 (46)	0.358	19 (59)	24 (65)	0.427
Vascular	13 (23)	5 (39)		9 (28)	9 (24)	
Other	5 (9)	1 (8)		2 (6)	4 (11)	
Intraoperative	1 (2)	1 (8)		2 (6)	0 (0)	
**Surgical Index complication**						
POPF	22 (39)	3 (23)	0.417	12 (38)	13 (35)	0.021
PPH	9 (16)	6 (46)		11 (34)	4 (11)	
Abdominal thrombosis	7 (13)	1 (8)		3 (9)	5 (14)	
DGE	7 (13)	0 (0)		1 (3)	6 (16)	
Anastomotic leakage *	5 (9)	1 (8)		1 (3)	5 (14)	
Intraoperative complication	1 (2)	1 (8)		2 (6)	0 (0)	
Others	5 (9)	1 (8)		2 (6)	4 (11)	
**Category of cause of death**						
Abdominal sepsis	34 (61)	9 (69)	0.083	20 (63)	23 (62)	0.010
Abdominal ischemia **	11 (20)	1 (8)		5 (16)	7 (19)	
Pulmonary sepsis	8 (14)	0 (0)		1 (3)	7 (19)	
Hemorrhagic shock	3 (5)	3 (23)		6 (19)	0 (0)	

POPF = post-operative pancreatic fistula, PPH = post-pancreatectomy hemorrhage, * except pancreatic anastomosis, ** including intestinal or hepatic ischemia.

**Table 5:** Distribution of questionnaire responses according to surgical parameters.

		Whipple/PPPD *n* = 68	Left Pancreatectomy *n* = 14	*p*-Value	Vascular Resection		*p*-Value	Multivisceral Resection		*p*-Value
					Yes *n* = 22	No *n* = 64		Yes *n* = 28	No *n* = 58	
**Time to death (days)**		34 [1–126]	30 [1–80]	0.885	37 [1–146]	33 [1–126]	0.850	38 [1–146]	33 [3–126]	0.736
**Classification of cause of death**										
Surgical		58 (85)	8 (57)	0.026	20 (91)	49 (77)	0.217	22 (79)	47 (81)	1.000
Non-surgical		10 (15)	6 (43)		2 (9)	15 (23)		6 (21)	11 (19)	
**Category of surgical index complication**										
Anastomotic		38 (66)	5 (63)	0.374	9 (45)	34 (69)	0.012	13 (59)	30 (64)	1.000
Vascular		14 (24)	1 (13)		10 (50)	8 (16)		6 (27)	12(26)	
Other		5 (9)	1 (13)		0 (0)	6 (12)		2 (9)	4 (9)	
Intraoperative		1 (2)	1 (13)		1 (5)	1 (2)		1 (5)	1 (2)	
**Surgical index complication**										
POPF		24 (41)	1 (13)	0.002	6 (30)	19 (39)	0.141	5 (23)	20 (43)	0.001
PPH		13 (22)	0 (0)		5 (25)	10 (20)		2 (9)	13 (28)	
Abdominal thrombosis		6 (10)	1 (13)		6 (30)	2 (4)		4 (18)	4 (9)	
DGE		7 (12)	0 (0)		2 (10)	5 (10)		2 (9)	5 (11)	
Anastomotic leakage*		2 (3)	4 (50)		0 (0)	6 (12)		6 (27)	0 (0)	
Intraoperative complication		1 (2)	1 (13)		1 (5)	1 (2)		1 (5)	1 (2)	
Others		5 (9)	1 (13)		0 (0)	6 (12)		2 (9)	4 (9)	
**Category of cause of death**										
Abdominal sepsis		36 (62)	7 (88)	0.518	8 (40)	35 (71)	0.012	14 (64)	19 (62)	0.347
Abdominal ischemia**		8 (14)	1 (13)		8 (40)	4 (8)		5 (23)	7 (15)	
Pulmonary sepsis		8 (14)	0 (0)		2 (10)	6 (12)		3 (14)	5 (11)	
Hemorrhagic shock		6 (10)	0 (0)		2 (10)	4 (8)		0 (0)	6 (13)	

**Table 6:** Test-retest reliability of the standardized review questionnaire.

Standardized Questionnaire Items	Answer Options	Peer Reviewn/ N (%)	Online Re-Reviewn/ N (%)	Cohen’s Kappa
**All cases**				
**Classification of cause of death?**	Surgical	14/20 (70)	13/20 (65)	0.886
	Non-surgical	6/20 (30)	7/20 (35)	
**Potential of improvement?**	Yes	15/20 (75)	13/20 (65)	0.765
	No	5/20 (25)	7/20 (35)	
**Surgical cause of death**				
**Category of index complication?**	Anastomotic	7/14 (50)	7/13 (54)	0.714
	Vascular	6/14 (43)	6/13 (46)	
	Other	1/14 (7)	0/13 (0)	
	Intraoperative	0/14 (0)	0/13 (0)	
**Index complication?**	POPF	6/14 (43)	4/13 (31)	0.492
	PPH	3/14 (21)	2/13 (15)	
	Abdominal thrombosis	3/14 (21)	4/13 (31)	
	DGE	0/14 (0)	0/13 (0)	
	Anastomotic leakage *	1/14 (7)	3/13 (23)	
	Intraoperative complication	0/14 (0)	0/13 (0)	
	Other	1/14 (7)	0/13 (0)	
**Category of cause of death?**	Abdominal sepsis	9/14 (64)	8/13 (6)	0.694
	Abdominal ischemia **	5/14 (36)	4/13 (31)	
	Pulmonary sepsis	0/14 (0)	0/13 (0)	
	Hemorrhagic shock	0/14 (0)	1/13 (8)	
**Non-surgical cause of death**				
**Index complication?**	Myocardial infarction	1/6 (17)	1/7 (14)	0.793
	Pulmonary embolism	2/6 (33)	2/7 (29)	
	Cerebral infarction	1/6 (17)	1/7 (14)	
	Heart insufficiency	1/6 (17)	2/7 (29)	
	Hypoxia	1/6 (17)	1/7 (14)	

POPF = post-operative pancreatic fistula, PPH = post-pancreatectomy hemorrhage, * except pancreatic anastomosis, ** including intestinal or hepatic ischemia.

**Table 7:** Inter-rater reliability between participants on standardized items of the online re-review.

Standardized Questionnaire Items	Answer Options	Fleiss’ Kappa
**All cases (*n* = 20)**		
**Classification of cause of death?**	Surgical	0.724
	Non-surgical	
**Potential of improvement?**	Yes	0.262
	No	
**Surgical cause of death (*n* = 13)**		
**Category of index complication?**	Anastomotic	0.455
	Vascular	
	Other	
	Intraoperative	
**Index complication?**	POPF	0.424
	PPH	
	Abdominal thrombosis	
	DGE	
	Anastomotic leakage *	
	Intraoperative complication	
	Others	
**Category of cause of death?**	Abdominal sepsis	0.533
	Abdominal ischemia **	
	Pulmonary sepsis	
	Hemorrhagic shock	

POPF = post-operative pancreatic fistula, PPH = post-pancreatectomy hemorrhage, * except pancreatic anastomosis, ** including intestinal or hepatic ischemia.

## Data Availability

All data presented in this study are available in the article.

## Abbreviations

POPF	Post-operative pancreatic fistula
PPH	Post-pancreatectomy hemorrhage
PPPD	Pylorus-preserving pancreaticoduodenectomy
